# SMARCA4-deficient thoracic tumor detected by [^18^F]FDG PET/CT

**DOI:** 10.1186/s41824-021-00102-5

**Published:** 2021-04-27

**Authors:** Tsubasa Okazaki, Kota Yokoyama, Jyunichi Tsuchiya, Takayuki Honda, Yuya Ishikawa, Susumu Kirimura, Yasunari Miyazaki, Ukihide Tateishi

**Affiliations:** 1 Department of Diagnostic Radiology, Tokyo Medical and Dental University, 1-5-45, Yushima, Bunkyo-ku, Tokyo, 113-8519 Japan; 2 Department of Respiratory Medicine, Tokyo Medical and Dental University, Tokyo, Japan; 3 Department of Thoracic Surgery, Tokyo Medical and Dental University, Tokyo, Japan; 4 Department of Comprehensive Pathology, Tokyo Medical and Dental University, Tokyo, Japan

**Keywords:** [^18^F]FDG, PET/CT, SMARCA4-deficient thoracic tumor

## Abstract

**Background:**

SMARCA4-deficient thoracic tumor (SMARCA4-DTT) is a distinct entity of undifferentiated thoracic malignancies newly introduced in 2015. Due to its unique clinical characteristic with aggressive thoracic tumor mostly observed in heavy smoker man with emphysema, with poor prognosis, many physicians are becoming increasingly aware of the disease; however, reports on 2-deoxy-2-[^18^F] fluoroglucose positron emission tomography/computed tomography ([^18^F]FDG PET/CT) have been limited; thus, this disease is not yet widely known to nuclear medicine clinicians. As a first step in discussing the usefulness of [^18^F]FDG PET/CT for this disease, we present a case in which [^18^F]FDG PET/CT played a clinically important role.

**Case:**

A 74-year-old heavy smoker man with an anamnesis of severe emphysema characterized by pleural thickening and abnormal enhancement in CT underwent 18F-FDG PET/CT for further examination. [^18^F]FDG-avid pleural nodules infiltrating into the chest wall were detected and pathologically diagnosed as SMARCA4-DTT with biopsy.

**Conclusion:**

SMARCA4-deficient thoracic tumor should be considered in a [^18^F]FDG-avid aggressive thoracic tumor in heavy smoker men with emphysema.

## Background

SMARCA4-deficient thoracic tumor (SMARCA4-DTT) is a distinct entity of undifferentiated thoracic malignancies newly introduced by Le Loarer et al. in 2015. SMARCA4-DTT is characterized by loss of SMARCA4, showing undifferentiated round cell or rhabdoid morphology presenting as aggressive behavior with compressive or infiltrative tumors mostly involving the mediastinum, lung, and/or pleura (Le Loarer et al. 2015; Crombé et al. 2019; Yoshida et al. 2017). Previous studies reported that it has male predominance (73.0–91.6%) and younger onset (median age 39–58, range 27–90 years), with a heavy smoking history (Le Loarer et al. 2015; Rekhtman et al. 2020; Perret et al. 2019; Yoshida et al. 2017). Because of its aggressive behavior and absence of effective therapies, it has a poor prognosis with reported median overall survival of 7 months (Le Loarer et al. 2015). Computed tomography (CT) usually shows relatively large tumor (mean size 9.2 [2.2–18.3] cm), showing an extensive involvement of thoracic structures, such as mediastinal, chest wall, and/or lung infiltration (Rekhtman et al. 2020; Crombé et al. 2019). Pulmonary emphysema/bullae are also common findings (Yoshida et al. 2017) due to the heavy smoking history, which could also be seen in other thoracic malignancies, such as lung cancer or malignant mesothelioma (Perret et al. 2019; Yoshida et al. 2017). Reports on its diagnosis using 2-deoxy-2-[^18^F]fluoroglucose positron emission tomography/computed tomography ([^18^F]FDG PET/CT) are limited; however, only a few studies reported it to show strong avidity to date (Rekhtman et al. 2020; Yoshida et al. 2017). Recently, the increasing number of reports with unique clinical characteristics made many physicians aware of the disease; however, due to the limited number of reports on [^18^F]FDG PET/CT, it is not yet widely known to nuclear medicine clinicians. Herein, we report an educational case of SMARCA4-DTT, showing high activity on [^18^F]FDG PET/CT that demonstrated chest wall infiltration, which was difficult to differentiate from other thoracic malignancies.

## Case presentation

**Fig. 1:**
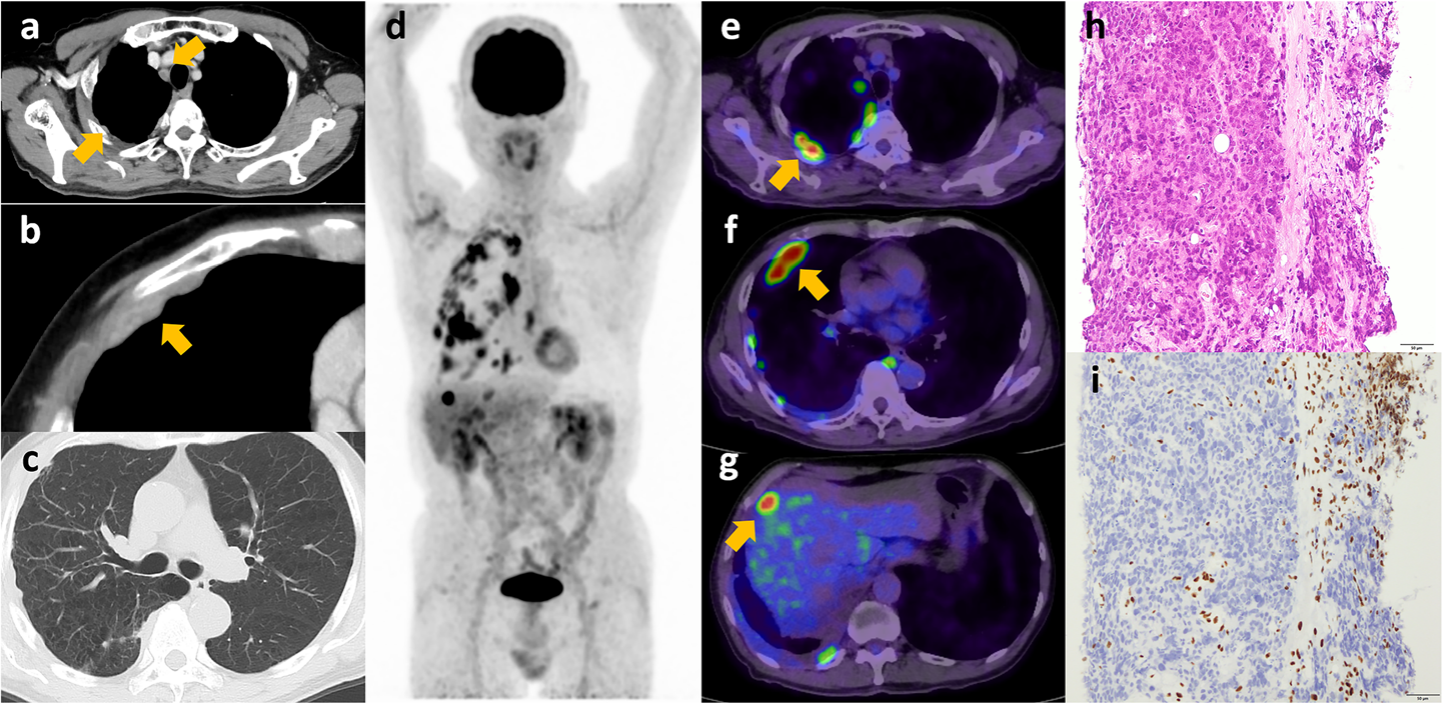
SMARCA4-deficient thoracic tumor detected by [^18^F]FDG PET/CT. **a** Contrast-enhanced CT shows pleural thickening with abnormal enhancement. **b** Infiltration into the right anterior chest wall of the lesion (yellow arrow) can be observed. **c** Background emphysema is seen. **d** Maximum intensity projection of PET image shows multiple [^18^F]FDG-avid nodules in right thoracic areas. **e**–**g** PET/CT fusion axial images showing multiple [^18^F]FDG-avid nodules located in the pleura infiltrating into the chest wall. **h** Hematoxylin–eosin stain of the tumor biopsy specimen shows dense proliferation of atypical epithelial-like cells. **i** SMARCA4 stain is negative in tumor cells leading to a diagnosis of SMARCA4-deficient thoracic tumor

A 74-year-old heavy smoker man with an anamnesis of severe emphysema presented with a 2-week history of right chest pain. He was diagnosed with mild pleural thickening 6 months before presentation, and contrast-enhanced CT revealed worsening of pleural thickening and abnormal enhancement infiltrating into the right anterior chest wall (Fig. 1; a, b: yellow arrows) with severe emphysema (c), resulting in a suspected diagnosis of malignant pleural mesothelioma.

Further examination with [^18^F]FDG PET/CT was performed. Images were acquired on a PET/CT scanner developed by Toshiba Medical Systems (Aquiduo and Celesteion; Tochigi, Japan) at 60 min after injecting 211 MBq of [^18^F]FDG. It revealed a marked maximal standardized uptake value of 9.6 in the lesions, suggesting a highly aggressive tumor (d–g: yellow arrows). CT-guided biopsy was performed, and pathological findings showed dense proliferation of atypical epithelial-like cells that also infiltrated into the chest wall (h) with the loss of SMARCA4 (i), resulting in the diagnosis of SMARCA4-DTT. Chemotherapy was initiated but resulted only in a temporary response, and the patient’s condition worsened in several months.

## Discussion

SMARCA4-DTT is a new entity first introduced in 2015 (Le Loarer et al. 2015), with only few reports demonstrating the use of [^18^F]FDG PET/CT for its diagnosis. Therefore, it was difficult to diagnose at the first visit. CT findings with pleural thickening and severe emphysema usually lead to the diagnosis of pleural plaque or malignant mesothelioma; however, in this case, [^18^F]FDG PET/CT played an important role in making us aware that the disease is a more aggressive tumor. In retrospect, chest wall infiltration and diffuse distribution of the tumor observed in [^18^F]FDG PET/CT appeared to be extremely fast for malignant mesothelioma. To the best of our knowledge, this is the first case report showing that [^18^F]FDG PET/CT may be useful for differentiating SMARCA4-DTT from malignant mesothelioma.

In a largest case series of 21 cases that reported imaging findings for this disease, four patterns of [^18^F]FDG PET/CT were proposed: mediastinal, pleural, cervical, and retroperitoneal (Crombé et al. 2019). The present case was close to the pleural pattern, but was different from previous reports because it did not form one large mass. Thus, this case is new in that respect; however, it appeared to show typical clinical findings. Since SMARCA4-DTT is often misdiagnosed as a carcinoma of unknown primary origin or as an undifferentiated carcinoma (Yoshida et al. 2017), nuclear medicine clinicians should include this disease in their list of differential diagnoses. To date, the majority of SMARCA4-DTTs have been reported as SMARCA4-deficient thoracic sarcomas; however, there are increasing reports of carcinomas. Since the present case was an undifferentiated tumor that was pathologically difficult to clearly classify as sarcoma or carcinoma, we described it as SMARCA4-DTT.

No definitive treatment has been established; however, several reports showed the effectiveness of molecular targeted therapies such as nivolumab (Iijima et al. 2020; Naito et al. 2019) or pembrolizumab (Henon et al. 2019; Takada et al. 2019) with patients showing remission. [^18^F]FDG PET/CT is expected to play an important role in the early diagnosis of this disease and will contribute to the improvement of its prognosis. Furthermore, the value of [^18^F]FDG PET/CT in therapeutic assessment should be investigated and established as with other malignant tumors (Becker et al. 2020; Unterrainer et al. 2020).

## Conclusion

We expect the present case helps nuclear medicine physicians to widely recognize this new disease and include SMARCA4-DTT as the differential diagnosis when encountering a [^18^F]FDG-avid aggressive thoracic tumor in heavy smoker men with emphysema. This report is also expected to establish strong evidence on the usefulness of [^18^F]FDG PET/CT for the diagnosis and treatment evaluation of this new disease entity.

## Data Availability

The images and biopsy samples remain available in our database.

## Abbreviations

SMARCA4-DTT: SMARCA4- deficient thoracic tumor
[^18^F]FDG: 2-Deoxy-2-[^18^F]fluoroglucose
PET: Positron emission tomography
CT: Computed tomography
